# Rationale & design of the PROMISES study: a prospective assessment and validation study of salivary progesterone as a test for preterm birth in pregnant women from rural India

**DOI:** 10.1186/s12978-018-0657-6

**Published:** 2018-12-22

**Authors:** Pankhuri Sharma, Simi Khan, Mohan Ghule, V. B. Shivkumar, Ritu Dargan, Paul T. Seed, Archana Sarkar, Sunil Mehra, Poonam Varma Shivkumar, Rachel M. Tribe

**Affiliations:** 1 Research and Innovation Unit, MAMTA Health Institute for Mother and Child, New Delhi, 110048 India; 2 Department of Pathology, Mahatma Gandhi Institute for Medical Sciences, Sevagram, Wardha, Maharashtra 442102 India; 3 Obstetrics and Gynecology, Independent Consultant, MAMTA Health Institute for Mother and Child, 110048, New Delhi, India; 4 Department of Women and Children’s Health, School of Life Course Sciences, King’s College London, St Thomas’ Hospital Campu, London, SE1 7EH UK; 5 Department of Obstetrics and Gynecology, Mahatma Gandhi Institute for Medical Sciences, Sevagram, Wardha, Maharashtra 442102 India

**Keywords:** Pregnancy, Spontaneous preterm birth, Low resource settings, Progesterone, Saliva, Neonatal mortality

## Abstract

**Background:**

In India, 3.6 million pregnancies are affected by preterm birth annually, with many infants dying or surviving with disability. Currently, there is no simple test available for screening all women at risk of spontaneous PTB in low income setting, although high resource settings routinely use cervical length measurement and cervico-vaginal fluid fetal fibronectin for identification and care of women at risk due to clinical history. In rural India, where the public health system has limited infrastructure, trained staff and equipment, there is a greater need to develop a low-cost screening approach for providing early referral, treatment and remedial support for pregnant women at risk of preterm birth. There is interest in the use of a salivary progesterone test as a screening tool preliminary evidence from India, Egypt and UK has shown promise for this type of test. The test requires further validation in a low resource community setting.

**Methods:**

The Promises study aims to validate and test the feasibility of introducing a low-cost salivary progesterone preterm birth prediction test in two rural districts in India with high rates of prematurity. It is a prospective study of 2000 pregnant women recruited from Panna and Satna in Madhya Pradesh over approximately 24 months. Demographic and pregnancy outcome data will be collected, and pregnancies will be dated by ultrasound sonography. Saliva progesterone will be measured by ELISA in samples obtained between 24–28 weeks of gestation. The association between salivary progesterone and preterm birth will be determined and the utility of salivary progesterone to predict preterm birth < 34, as well as < 30 and < 37 weeks assessed. Additional qualitative data will be obtained in terms of acceptability and feasibility of saliva progesterone testing and knowledge of PTB.

**Discussion:**

A validated cost-effective saliva test, which has potential for further adaptation to a ‘point of care’ setting will allow early identification of pregnant women at risk of preterm birth, who can be linked to an effective pathway of care and support to reduce preterm birth and associated adverse consequences. This will reduce both economic and emotional burden on the affected women and their families**.**

## Plain English summary

In India, many babies are born too early in pregnancy (a premature birth that occurs 3 weeks or earlier than term birth which is 40 weeks of pregnancy). Many premature babies die at birth but those who survive also can suffer health issues and disability. Doctors would like to be able to test all women in early pregnancy to determine their risk of a premature birth so that they can provide suitable advice and care, but such a test does not yet exist. In rural situations where healthcare is hard to access, a test that could identify which pregnancies are most at risk would be useful for advising women about pregnancy care and their plans for labour and birth. One potential test is the measurement of the pregnancy hormone progesterone in saliva. Saliva can be obtained easily and safely from women. There is some research that indicates that this test might be useful, but it requires further research before it can be used routinely by doctors or midwives. This article describes the Promises study which will collect pregnancy and birth information from 2000 pregnant women in rural India (two districts in Madhya Pradesh). Each woman will have her pregnancy accurately dated using an ultrasound scan and be asked to provide a saliva sample to measure progesterone between 24 and 28 weeks of pregnancy. The results will be analysed to assess whether the level of progesterone in saliva can identify women who are going to have a baby too early.

## Background

The 2012 Global Action Report on Preterm Birth (PTB) estimates that 15 million PTBs occur annually and that one million neonatal deaths result from prematurity related causes each year [1]. There are 12.9 million premature births annually worldwide [2]. Approximately, over 60% of preterm births occur in in Sub-Saharan Africa and south Asia. PTB, especially early PTB (< 34 weeks of gestation) [3], is a major cause of neonatal mortality and contributes to 75% of neonatal morbidity and 70% of neonatal mortality in industrialized countries [4]. In children under five, preterm birth is the second largest direct cause of child deaths [3]. Preterm babies, in addition to being at a higher risk of neonatal mortality, are at an increased risk of post-neonatal mortality, stunting, and long-term neuro-developmental impairment during childhood. For babies who survive, there is an increased risk of disability, which exacts a heavy load on families and health systems [5]. An estimated 70% of PTBs results from idiopathic preterm labour or pre-labour rupture of membranes and the remainder are iatrogenic because of obstetric or medical indications [6]. PTB has high economic and social burden in terms of health care needs, emotional stress on family in case of preterm baby death and hospital stay [7] (WHO 2012). India has the highest number of PTBs and preterm deaths worldwide [5], contributing to 25% of the overall global preterm related death; of a total 27 million babies born annually, 3.6 million babies are born preterm, and over 300,000 of these preterm babies die each year because of associated complications [5, 8]. This is even more challenging in Indian rural setting where most women are unaware of the risk of PTB; and do not readily access available care and support. This leads to increased maternal and neonatal morbidity and mortality in rural India [9]. A systematic analysis of global, regional and national causes of child mortality in 2013 identified PTB birth complications and infections to be the two major causes of neonatal deaths in India [10]. In addition, surviving preterm babies are at greater risk for short-term health complications including acute respiratory, gastrointestinal, infectious, central nervous system, hearing, and vision problems, and long- term neuro-developmental disabilities such as cerebral palsy, impaired learning and visual disorders, as well as chronic diseases in adulthood [11, 12].

Addressing PTBs is essential in order to progress on Sustainable Development Goal (SDG) 3, to reduce the neonatal mortality rate (NNMR) to as low as 12 per 1000 live birth [13] especially in countries with high burden of PTB. Despite the magnitude of the problem, there is no established early pregnancy-screening test or effective treatment for women once a high risk of spontaneous preterm birth (sPTB) is ascertained [14].

In high resource settings, there is the option for mid-trimester risk prediction using transvaginal ultrasound measurement of cervical length measurement and cervico-vaginal fetal fibronectin [14–18] for identification and care of women who are already deemed to be at risk due to clinical history (previous PTB, previous mid-trimester miscarriage or premature rupture of membranes). These tests for predicting PTB risk are invasive, expensive, require skilled workforce and facilities and are not feasible for rural Indian setting where such facilities are largely unavailable or scarce. Development of an acceptable, non-invasive, ‘point of care’ test for accurate prediction of women at risk of early PTB has considerable potential to reduce infant deaths in low-resource settings through improvements in antenatal and postnatal care uptake and or by referral of high risk women to higher level health facilities with experience in care of vulnerable infants. A small study in the UK has indicated that salivary progesterone concentrations are significantly lower from 24 weeks of pregnancy in women, who had a spontaneous PTB (< 34 weeks gestation) [19]. This highlighted the potential of using salivary progesterone measurements for the prediction of early preterm labour and determining specific women that might benefit from intervention [12, 20–25]. Subsequently, two further studies have confirmed the predictive potential of salivary progesterone measurements in urban populations in India (high risk women) [26] and Egypt [27] (high risk and low risk women). Both indicated that salivary progesterone can identify women destined to deliver < 34 weeks with a sensitivity, specificity, positive and negative prediction values of 83–84%; 86–90%, 60–89.8% and 85.9–95% respectively. With this backdrop, our study aim is to assess and validate the use of a non-invasive salivary progesterone test in a community setting where there is limited or no routine intervention for PTB, to obtain definitive evidence to support the widespread introduction of salivary progesterone testing for PTB in India.

## Methods/design

### Hypothesis and aims

In India, there is a clear need to introduce some form of antenatal screening for PTB if there is to be a reduction of PTB and associated neonatal mortality. Saliva testing of progesterone, which reflects the unbound, unconjugated biologically active fraction of the plasma hormone level profile, has benefits over using a more invasive blood test. We hypothesised that successful validation of the salivary progesterone test could provide an inexpensive, easy to use, non-invasive and acceptable test for early detection of risk of PTB among pregnant women in rural India.

### Study objectives

1. To determine the association and assess the performance of a salivary progesterone test (specificity, sensitivity, predictive value and ROC) for prediction of PTB risk at < 34 weeks and < 37 weeks of gestation.
2. To assess the feasibility, and acceptability to women and health care workers, of this test in a rural setting.

As part of objective two, the goal is to gain a fuller understanding of the range of facilitating factors and barriers influencing the use of salivary progesterone PTB tests in Indian rural settings.

### Study sites

**Fig. 1:**
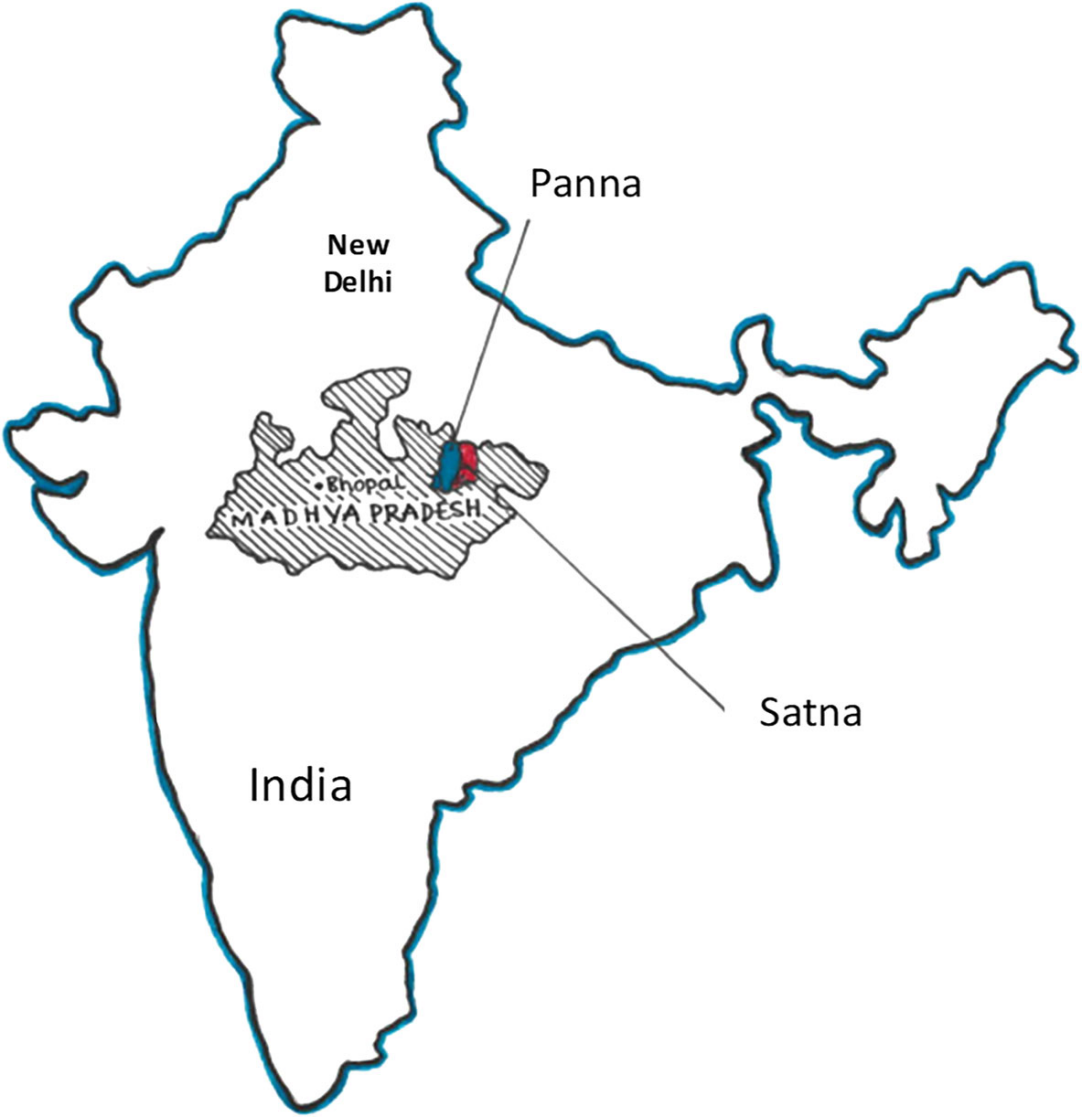
Location of study districts (Panna and Satna) in the state of Madhya Pradesh, India

This study will be carried out in the state of Madhya Pradesh, India (Fig. 1). The NNMR of Madhya Pradesh is 34, which is quite higher than the national average [28]. Two districts (Panna and Satna) with high fertility (Crude Birth Rate: 31.3 and 28.2respectively) and high rates of NNMR (61–57 per 1000 live births) [29] particularly resulting from births < 34 weeks [30] and an infant mortality rate of 85 per 1000 live births were selected. Major contributors to PTB/neonatal mortality [21] in these tribal dominated districts are poverty, physical stress, malnutrition, poor public health facilities and low health service utilization. Two blocks each covering more than 100,000 populations will be included from each district.

### Study design

**Fig. 2:**
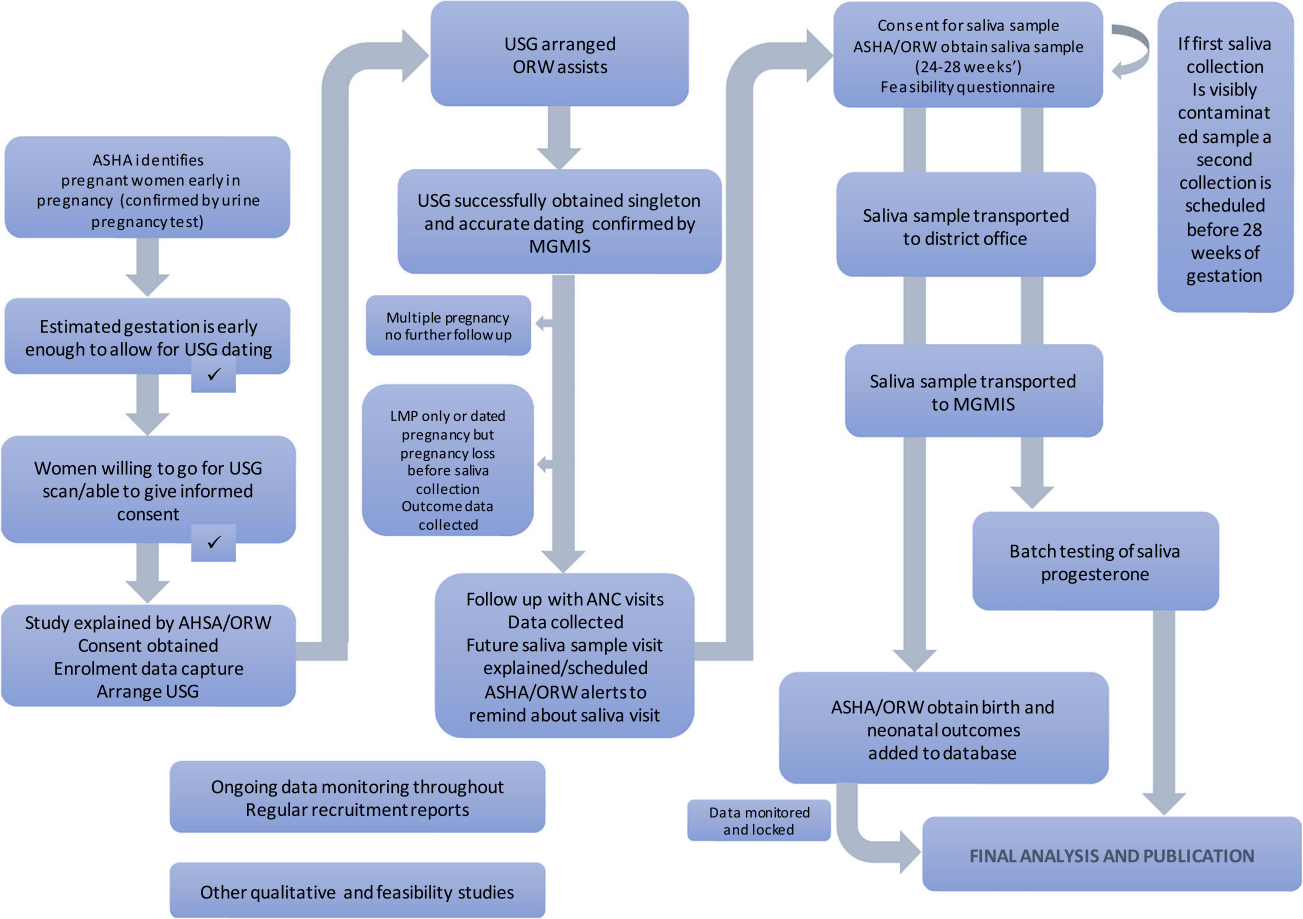
Process Flow Chart of the Project

A prospective observational cohort study to recruit 2000 pregnant women from the two districts of Madhya Pradesh (Fig. 1). The flow of participants through the study is shown in Fig. 2. Mapping was conducted by the field staff to identify areas that had comparable population density and somewhat comparable geographic size, with natural boundaries noted.

Based on the PTB rates in two study districts (~ 23%), it was estimated that recruitment of 2000 women would identify ~ 460 preterm deliveries at < 37 weeks’, providing ample power to predict PTB at this gestation. Evidence suggests that the salivary progesterone test works best to detect birth < 34 weeks [19], but the incidence for this gestation is not known for the study districts. However, if 189 cases of PTB < 34 weeks’ are identified (9.5% incidence), this would provide 90% power to distinguish 80% from 70% sensitivity. Pregnant women will be identified and consented early in pregnancy so that an accurate dating scan can be achieved, and baseline obstetric history and demographic data obtained. Women will then be asked on a subsequent visit to provide a saliva sample between 24 and 28 weeks of pregnancy. Women will be followed-up till delivery for birth outcomes.

### Study duration

The study will be conducted for a period of 24 months. The major activities to be conducted include project planning, gaining of ethical approval, training of frontline functionaries and laboratory technicians, line listing and recruitment of pregnant women, salivary sample collection, storage and transportation and batch analysis of saliva progesterone.

### Inclusion & Exclusion criteria at time of recruitment

Inclusion Criteria:

1. Women whose pregnancy is confirmed will be registered.

2. Women with singleton pregnancy.

3. Pregnant women whose gestation period is established by ultrasound scan.

Exclusion criteria:

1. Pregnant women unwilling or unable to give informed consent
2. Women unable to give informed consent
3. Pregnancy not dated by ultrasound (or by late ultrasound > 20 weeks).
4. Failure to confirm viable intrauterine pregnancy by ultrasound scan
5. Women with multiple pregnancy

Demographic and pregnancy outcome data will be retained for women whose pregnancies are only dated by last menstrual period as this will still contribute to understanding of clinical factor and PTB.

At the time of saliva collection, women are consented for the provision of a saliva sample for progesterone analysis. Women unable or unwilling to provide a saliva sample will be retained in the study for clinical data collection.

### Ethics committees review and approval

Ethical approval was granted by the MAMTA Health Institute for Mother and Child (MAMTA-HIMC) Ethical Review Board (MERB/Dec-2016/002) and the Institutional Review Board of Mahatma Gandhi Institute of Medical Science (MGIMS/IEC/OBGY/289/2016). As the project included sample analysis, written (Hindi or English), signed (or with thumb impression, if illiterate) and informed consent is required from each eligible pregnant mother both at study entry and the time of salivary sample collection.

A confidential ID will be given to each participant and inserted in consent forms, test forms, questionnaires and sample collected for the purpose of data linkage and analysis.

### Training and knowledge of field staff

Before the recruitment of participants and sample collection, training involving practical sessions will be provided for study staff including district coordinators and outreach workers and the laboratory teams responsible for saliva sample collection and saliva testing (sample storage and immunoassay at the health facility).

### Research tools

A package of research tools has been developed, translated in Hindi language and pre-tested for data collection. The research tools consisting of structured questionnaires and semi-structured in-depth interviews are: a) A tracking tool designed for recruited pregnant women to obtain information on their demographic details, and previous pregnancies and follow their current pregnancy including antenatal care visits, Ultra sonography (USG) scan details and post-natal tracking for delivery outcomes along with documentation of saliva sample collection, storage and transportation details, analysis process and progesterone results. b) Pre-post training assessment tool for frontline functionaries to determine the effectiveness of training on the knowledge on preterm birth. c) In-depth interview (for pregnant women and frontline functionaries on knowledge of preterm birth, accessibility to relevant antenatal care, and the perception of the test and its usefulness.

### Recruitment and consent process of pregnant women

Women will be identified as early in pregnancy as possible (ideally < 12 weeks’ of gestation). The list of eligible pregnant women will be obtained from the local Accredited Social Health Activist (ASHA) workers and the local awareness campaigns by the study’s outreach workers (ORW). They will establish rapport with the pregnant women and family members. The outreach workers will provide information to pregnant women about pregnancy health care, PTB and the importance of PTB screening. Any woman appearing as needing medical attention will be encouraged to attend their local health facility by the ORW.

All participants will be provided with a participant information sheet (PIS) with verbal translation available for local language speaking women. Women will be consented by an appropriately trained study staff (i.e. district coordinator or outreach worker). They will be provided information about the study and will be allowed adequate time to read the participant information sheet and provide written informed consent. Where there is doubt about the ability of a volunteer to provide informed consent, they will not be recruited. In case of potential recruits have impaired hearing, the outreach workers (ORW) along with frontline workers will make every effort to ensure that her answers to any queries are fully understood and may consider asking a member of the family to attend the discussion prior to the signing of the consent form. In case of illiterate women or the women with inability to understand the concept of informed consent the outreach workers would read the information sheet to the volunteer in the presence of a literate witness which may be the frontline workers (ASHAs), preferably. The consent form should also be read to the literate witness prior to signing or making a mark and countersigned by the literate witness. The consent form will be filed in the participants’ consent form file in study number order and stored in a locked filing cabinet at the District Outreach office separated from study data.

### Initial contact with potential participants

For all the women who agreed for participation, information will be collected on demographic profile (age, education, religion, caste, occupation, family type, per capita income etc.); reproductive phase with respect to age at marriage, occurrence of live birth, place of delivery, number of antenatal care and post-natal visits, post-natal complications, if any etc. Participants will undertake USG dating scans (ideally before 14 weeks of pregnancy) and baseline obstetric history and demographic data will be obtained. They will also be informed that during a subsequent visit they would be requested to provide saliva sample between 24 and 28 weeks of pregnancy, and that they will be followed-up in the postnatal period to obtain birth outcomes.

Each pregnant woman recruited in the study will be provided a Unique ID at the time of registration. Link anonymised data will be entered onto a study specific internet-based database (MedSciNet https://promises.medscinet.com/). The database is password protected and will have different level of users (i.e. unit users, monitoring users and global administrators).

### Arranging the first household visit

The first household visit will include consent and recruitment of the pregnant women for the study. This will also include briefing of the women about the importance of the study and the negative outcomes for mother and baby relating to preterm birth. Inclusion/exclusion criteria will be checked thoroughly. Any queries from the participants will be addressed.

### Antenatal care (ANC) visit (for ultrasonography)

Recruited pregnant women will be taken to an identified government hospital facilities or private facility for ultrasound dating of pregnancy. The radiologist at this facility will record fetal measurements and preserve data the frozen scan frames (anonymised labelled with study ID and date only). A consultant radiologist at MGIMS will provide field-based training and randomly check data/images as a second verification step to determine agreed estimated date of delivery. Anticipating that at scan, a proportion of pregnancies will be found to be > 14 weeks, a range of fetal measurements will be taken (crown-rump length, bi-parietal diameter; head circumference; abdominal circumference or diameter to include second trimester scans [31].

### Visit for sample collection

A further visit to participants by outreach workers to collect the saliva sample will occur between 24 and 28 weeks of gestation of the participants. At this stage, an additional informed consent will be gained.

### Sample collection procedure

Women will be encouraged to provide saliva sample after an overnight fasting. Sample collection within 60 min after eating a major meal or within 12 h after consuming alcohol will be strictly avoided. Acidic or high sugar foods can compromise assay performance by lowering sample pH and influencing bacterial growth. To minimize these factors, participants will be asked to rinse mouth thoroughly with water 10 min before sample is collected. Saliva will be collected by the passive drool method into a small. The start time will be noted, and a sample of at least 5 ml of saliva will be collected into graduated labelled polypropylene container over approximately 5–10 min and then placed into ice. Saliva samples visibly contaminated with blood will be discarded and recollected where possible within the 7 days.

### Sample transportation and storage

The unique Patient ID will be used (along with other time and date information) to track sample collection and storage. Samples will be stored at the district field office at − 20 °C until dispatched to the MGIMS laboratory. The time of saliva collection and the time at which samples reached freezer is noted at this time. These data along with bar coded tube information will be entered into the participant’s electronic record form on the database.

#### I. Transportation from study site to field office

Although progesterone is relatively stable, due to the high temperature that can be experienced at the study site, after collection of the saliva sample from a participant, it will immediately be placed inside the ice box. The samples will be transferred within 30 min to district field office. All the samples in district office will be kept in − 20 °C.

#### II. Transportation from field office to MGMIS laboratory

Frozen samples will be transferred using cold (4 °C) transportation carriers and will be sent to the MGMIS laboratory for progesterone ELISA analysis in batches. The district coordinators will undertake the activity to avoid loss of samples during transition. This approach includes a planned freeze-thaw cycle to ensure that samples will arrive cold (temperature monitored on arrival; thawed samples will be divided into two aliquots (barcode labelled) for storage at − 80 °C and future analysis of progesterone by ELISA (Demeditec Diagnostics GmbH, CAT No. DES 6633).

### Follow up visit

The recruited participants of the study will be visited to collect information about pregnancy outcome (e.g. time and date of delivery, gestation at delivery, medications received and total inpatient nights) and neonatal outcome (e.g. male or female, live/dead, birth weight, congenital abnormality, any neonatal complications and date of discharge). Additional/missing details of any pregnancy complications will be tracked through their registered antenatal care visits cards.

### Exit from the study

The delivery outcome information will be obtained during the final visit by ORWs from all the registered women in the study (i.e. also including women who did not get a USG, were outside the study gestation window, or did not provide saliva).

### Data management

A data safety monitoring board (DSMB), responsible for data management, reporting, protocol sharing and regular reviews, was convened with members from the three collaborating institutions (MGIMS, MAMTA and Kings College London).

### Study outcomes

Three major outcomes will be determined.

i. The association between salivary progesterone at 24–28 weeks of pregnancy and PTB.

ii. Performance of salivary progesterone test (sensitivity, specificity, predictive values and ROC areas) will be produced for different cut-off values of salivary progesterone for prediction of PTB (< 34 and < 37 weeks of gestation).

iii. Evidence of feasibility and acceptability of this low cost innovation for early identification of pregnant women in rural low resource setting.

For preterm birth, the primary endpoint will be spontaneous preterm birth or PPROM < 34 weeks and secondary endpoints < 37 weeks of pregnancy, < 30 weeks. Additional secondary endpoints will include gestation at delivery and a range of maternal and neonatal outcomes (composite and individual.

A qualitative approach will be used to determine the acceptability of the salivary progesterone test to pregnant women and frontline functionaries (qualitative interviews).

### Sample size and data analysis

A significant difference between cases of PTB and controls is not sufficient to show that a test is clinically useful. Both sensitivity and specificity must be evaluated. Accurately determining the sensitivity is harder (due to smaller numbers), and the sample size determination accordingly concentrates on this. Based on the PTB rates in two study districts (~ 23%), recruitment of 2000 women would potentially identify 460 preterm deliveries at < 37 weeks’ providing ample power to predict PTB at this gestation and to use data for discovery and validation. Data from Lachelin et al., [19] indicates the salivary progesterone test works best to detect birth < 34 weeks, but this incidence for this gestation is not known for the study districts. However, if 189 cases of PTB < 34 weeks’ are identified (9.5% incidence), this would provide 90% power to distinguish 80% from 70% sensitivity. Calculations were carried out in Stata version 13.1 using the command ‘sampsi’ (StataCorp, College Station, Texas).

At study completion, data will be downloaded into a statistical package in the statistical package Stata, version 15 or later, (StataCorp) and additional data cleaning checks applied. Clinical and pregnancy outcome data will be described for the whole cohort.

Additional predefined exclusion criteria relating to sample availability and contamination, and pregnancy outcome (e.g. iatrogenic PTB, maternal medications such as antidepressants and progesterone, use of cervical cerclage or Arabin pessary).

The data will be randomly split into training and validation sets, each containing the same number of cases (premature deliveries before 37 and 34 weeks) and controls (term deliveries). The training set will be used to determine the best cut-offs to use for a rule-in and a rule-out test. Based on Priya et al., [26], a limited number of simple cut-offs will be investigated: 500, 1000, 1500, 2000, 2500, 3000 pg/ml. These results will define two thresholds, allowing women to be triaged into three groups: “green” (salivary progesterone above the upper threshold), “amber” (progesterone between the two thresholds) and “red” (progesterone below the lower threshold). It is envisaged that if the test proves suitable for general use, the “green” group will receive usual care; the “amber” group will receive more intense advice about meaning of dating scan, due date, etc.; and the “red” group will be advised to change their birthing plan to include delivery in a district tertiary healthcare centre with a neonatal intensive care facility. Following STARD guidelines [32] for diagnostic tests, measures of test performance (sensitivity, specificity, predictive values and ROC areas) will be produced with 95% confidence intervals for the two cut-offs selected as predictors of the primary endpoint: PTB < 34 weeks of gestation, the secondary endpoints: PTB < 37 weeks’ and < 30 weeks’, and other important outcomes: perinatal and maternal and neonatal death and associated morbidities.

Qualitative data will be analysed following COREQ [33] standards. All the qualitative data collected through in-depth interviews will be translated into English transcripts. The analysis of the transcripts will be done using inductive approach. Themes will be identified during the review of the transcripts manually and further these themes will be grouped into key categories.

## Discussion

The protocol described includes adaptations that were implemented early in the study to address the operational challenge of ensuring timely USG. Initially, it was planned to only include women who had a USG dating pregnancy scan performed by 13^+ 6^ weeks of gestation. However, it was clear that the reported self-reported LMP, which was used to inform recruitment, and USG schedules were very unreliable mainly because of the social taboo associated with pregnancy disclosure in the first and second trimester. Following discussion, review of the literature and inclusion of additional USG measurement parameters [31], the window for USG dating was increased, with a focus on ensuring women were scanned by 16 weeks’ for the saliva testing if possible. This was decided to be a pragmatic approach for the study setting, and indeed, if the test proves to work, the revised research criteria would better reflect real-life conditions for implementation.

In India, the Maternal, Newborn, Child and Adolescent Health (RMNCH+A) strategic framework of 2013 laid out a vision and a plan for the country to end preventable newborn deaths, accelerate progress, and scale up high-impact yet cost-effective interventions. The successful validation of a cost effective, simple to use tool, with a predictor value of 70–80% for PTB, will allow easy identification of pregnant women who are at risk of PTB, who can be directed effectively to an appropriate level of care. Moreover, the Government of India has pledged to improve its neonatal mortality with a strong political will to achieve it. The India Newborn Action Plan (INAP) [34] is India’s committed response to the Global Every Newborn Action Plan (ENAP), launched in June 2014 at the 67th World Health Assembly, and Reproductive, One of the specific goals of INAP is ‘Ending Preventable Newborn Deaths to achieve “Single Digit NMR” by 2030, with all the states to individually achieve this target by 2035’. Although, the intervention package ‘Antenatal Screening for high risk pregnancies/complications and their management’ has shown considerable impact [35] on the prevention of many of the newborn deaths, stillbirths, and maternal deaths, it does not include early diagnosis/screening of PTB, which is the major cause of newborn deaths in India. The salivary progesterone test, if successful, will fill in the gap of providing a simple diagnostic tool for predicting PTB which can be easily adopted within the routine pregnancy/antenatal period at the level of community health workers and equip health system with an appropriate management plan for at-risk women for the rural settings. However, in tandem, it will require adequate USG facilities in these areas and increased education of women so that they reveal pregnancy earlier and access USG for accurate pregnancy dating. The future scale-up of the test, if successfully combined with accurate pregnancy dating, could lead significant improvements in care for pregnant women and neonatal outcomes related to PTB.

## Abbreviations

ANC: Antenatal care
ASHA: Accredited Social Health Activist
MAMTA-HIMC: Mamta Health Institute for Medical Sciences
MGIMS: Mahatma Gandhi Institute for Medical Sciences
NNMR: Neo natal mortality rate
ORW: Outreach Worker
PTB: Preterm Birth
USG: Ultra sonography

## References

[1] Howson CP, Kinney MV, McDougall L, Lawn JE: Born toon soon: preterm birth matters. Reprod Health 2013, 10(Suppl 1):S1-S1. 10.1186/1742-4755-10-S1-S1 PMC3828581 24625113

[2] Christianson A, Howson CP, Modell B: March of Dimes: global report on birth defects, the hidden toll of dying and disabled children. March of Dimes: global report on birth defects, the hidden toll of dying and disabled children 2005.

[3] Blencowe H, Cousens S, Chou D, Oestergaard M, Say L, Moller AB, Kinney M, Lawn J (2013). Born too soon: the global epidemiology of 15 million preterm births. Reprod Health.

[4] Wen SW, Smith G, Yang Q, Walker M (2004). Epidemiology of preterm birth and neonatal outcome. Semin Fetal Neonatal Med.

[5] Howson CP KM, Lawn JE, editor. Geneva: World Health Organization; 2012: March of Dimes, PMNCH, Save the Children, WHO. Born Too Soon: The Global Action Report on Preterm Birth. In*.*: World Health Organization; 2012.

[6] Goldenberg RL, Culhane JF, Iams JD, Romero R (2008). Epidemiology and causes of preterm birth. Lancet (London, England).

[7] Althabe F (2012). Born too soon: the global action report on preterm birth: World Health Organization.

[8] Blencowe H, Cousens S, Oestergaard MZ, Chou D, Moller A-B, Narwal R, Adler A, Vera Garcia C, Rohde S, Say L (2012). National, regional, and worldwide estimates of preterm birth rates in the year 2010 with time trends since 1990 for selected countries: a systematic analysis and implications. Lancet.

[9] Sankar MJ, Neogi SB, Sharma J, Chauhan M, Srivastava R, Prabhakar PK, Khera A, Kumar R, Zodpey S, Paul VK (2016). State of newborn health in India. J Perinatol.

[10] Liu L, Oza S, Hogan D, Perin J, Rudan I, Lawn JE, Cousens S, Mathers C, Black RE (2015). Global, regional, and national causes of child mortality in 2000-13, with projections to inform post-2015 priorities: an updated systematic analysis. Lancet.

[11] Institute of Medicine Committee on Understanding Premature B, Assuring Healthy O: The National Academies Collection: Reports funded by National Institutes of Health. In: *Preterm Birth: Causes, Consequences, and Prevention.* edn. Edited by Behrman RE, Butler AS. Washington (DC): National Academies Press (US) National Academy of Sciences.; 2007. 20669423

[12] Romero R, Conde-Agudelo A, Da Fonseca E, O’Brien JM, Cetingoz E, Creasy GW, Hassan SS, Nicolaides KH (2018). Vaginal progesterone for preventing preterm birth and adverse perinatal outcomes in singleton gestations with a short cervix: a meta-analysis of individual patient data. Am J Obstet Gynecol.

[13] SDGs U: United Nations Sustainable Development Goals. In*.*; 2015.

[14] Hezelgrave NL, Abbott DS, Radford SK, Seed PT, Girling JC, Filmer J, Tribe RM, Shennan AH (2016). Quantitative fetal fibronectin at 18 weeks of gestation to predict preterm birth in asymptomatic high-risk women. Obstet Gynecol.

[15] Watson HA, Carter J, Seed PT, Tribe RM, Shennan AH (2017). The QUiPP app: a safe alternative to a treat-all strategy for threatened preterm labor. Ultrasound Obstet Gynecol.

[16] Vandermolen BI, Hezelgrave NL, Smout EM, Abbott DS, Seed PT, Shennan AH: Quantitative fetal fibronectin and cervical length to predict preterm birth in asymptomatic women with previous cervical surgery. Am J Obstet Gynecol 2016, 215(4):480.e481–480.e410. 10.1016/j.ajog.2016.05.020 27267388

[17] Abbott DS, Radford SK, Seed PT, Tribe RM, Shennan AH (2013). Evaluation of a quantitative fetal fibronectin test for spontaneous preterm birth in symptomatic women. Am J Obstet Gynecol.

[18] Abbott DS, Hezelgrave NL, Seed PT, Norman JE, David AL, Bennett PR, Girling JC, Chandirimani M, Stock SJ, Carter J (2015). Quantitative fetal fibronectin to predict preterm birth in asymptomatic women at high risk. Obstet Gynecol.

[19] Lachelin GC, McGarrigle HH, Seed PT, Briley A, Shennan AH, Poston L (2009). Low saliva progesterone concentrations are associated with spontaneous early preterm labour (before 34 weeks of gestation) in women at increased risk of preterm delivery. Bjog.

[20] Hezelgrave NL, Watson HA, Ridout A, Diab F, Seed PT, Chin-Smith E, Tribe RM, Shennan AH (2016). Rationale and design of SuPPoRT: a multi-Centre randomised controlled trial to compare three treatments: cervical cerclage, cervical pessary and vaginal progesterone, for the prevention of preterm birth in women who develop a short cervix. BMC Pregnancy and Childbirth.

[21] Saccone G, Maruotti GM, Giudicepietro A, Martinelli P (2017). Effect of cervical pessary on spontaneous preterm birth in women with singleton pregnancies and short cervical length: a randomized clinical trial. Jama.

[22] Alfirevic Z, Owen J, Carreras Moratonas E, Sharp AN, Szychowski JM, Goya M (2013). Vaginal progesterone, cerclage or cervical pessary for preventing preterm birth in asymptomatic singleton pregnant women with a history of preterm birth and a sonographic short cervix. Ultrasound Obstet Gynecol.

[23] Cabrera-García L, Cruz-Melguizo S, Ruiz-Antorán B, Torres F, Velasco A, Martínez-Payo C, Avendaño-Solá C (2015). Evaluation of two treatment strategies for the prevention of preterm birth in women identified as at risk by ultrasound (PESAPRO trial): study protocol for a randomized controlled trial. Trials.

[24] Alfirevic Z, Stampalija T, Medley N (2017). Cervical stitch (cerclage) for preventing preterm birth in singleton pregnancy. Cochrane Database Syst Rev.

[25] Conde-Agudelo A, Romero R, Nicolaides K, Chaiworapongsa T, O'Brien JM, Cetingoz E, da Fonseca E, Creasy G, Soma-Pillay P, Fusey S (2013). Vaginal progesterone vs. cervical cerclage for the prevention of preterm birth in women with a sonographic short cervix, previous preterm birth, and singleton gestation: a systematic review and indirect comparison metaanalysis. Am J Obstet Gynecol.

[26] Priya B, Mustafa MD, Guleria K, Vaid NB, Banerjee BD, Ahmed RS (2013). Salivary progesterone as a biochemical marker to predict early preterm birth in asymptomatic high-risk women. Bjog.

[27] Maged AM, Mohesen M, Elhalwagy A, Abdelhafiz A (2015). Salivary progesterone and cervical length measurement as predictors of spontaneous preterm birth. J Matern Fetal Neonatal Med.

[28] Office of The Registrar General & Census Commissioner, India, Ministry Of Home Affairs Government of India Sample Registration Sample (SRS) Statistical Report 2015. http://www.censusindia.gov.in/vital_statistics/SRS_Report_2015/8.Chap%204-Mortality%20Indicators-2015.pdf.

[29] Ministry of Home Affairs GoI: Annual Health Survey 2012–13, Fact Sheet. In*.* Edited by Vital Statistics Division OotRGCC, India. New Delhi.

[30] The L (2012). A timely arrival for <em>born too soon</em>. Lancet.

[31] Salomon LJ, Alfirevic Z, Berghella V, Bilardo C, Hernandez-Andrade E, Johnsen SL, Kalache K, Leung KY, Malinger G, Munoz H (2011). Practice guidelines for performance of the routine mid-trimester fetal ultrasound scan. Ultrasound Obstet Gynecol.

[32] Cohen JF, Korevaar DA, Altman DG, Bruns DE, Gatsonis CA, Hooft L, Irwig L, Levine D, Reitsma JB, De Vet HC, Bossuyt PM. STARD 2015 guidelines for reporting diagnostic accuracy studies: explanation and elaboration. BMJ open. 2016;6(11):e012799. 10.1136/bmjopen-2016-012799 PMC5128957 28137831

[33] Tong A, Sainsbury P, Craig J (2007). Consolidated criteria for reporting qualitative research (COREQ): a 32-item checklist for interviews and focus groups. Int J Qual Health Care.

[34] India Newborn Action Plan (INAP), Child and Health Division, Ministry of Health and Family Welfare, Government of India; September 2014. http://nhm.gov.in/images/pdf/programmes/inap-final.pdf.

[35] Lawn JE, Blencowe H, Oza S, You D, Lee AC, Waiswa P, Lalli M, Bhutta Z, Barros AJ, Christian P, Mathers C. Every Newborn: progress, priorities, and potential beyond survival. The Lancet. 2014;384:189-205. 10.1016/S0140-6736(14)60496-7 24853593

